# P-1094. Real-World Experience with Maribavir for the Treatment of Cytomegalovirus Infection in Patients with Hematologic Malignancies

**DOI:** 10.1093/ofid/ofae631.1282

**Published:** 2025-01-29

**Authors:** Marilyne Daher, Fareed Khawaja, Amy Spallone, Terri Lynn Shigle, Ella Ariza Heredia, Micah M Bhatti, Roy F Chemaly

**Affiliations:** Baylor College of Medicine, Houston, Texas; The University of Texas MD Anderson Cancer Center, Houston, Texas; University of Texas MD Anderson Cancer Center, Houston, Texas; The University of Texas MD Anderson Cancer Center, Houston, Texas; The University of Texas MD Anderson Cancer Center, Houston, Texas; The University of Texas MD Anderson Cancer Center, Houston, Texas; University of Texas MD Anderson Cancer Center, Houston, Texas

## Abstract

**Background:**

Cytomegalovirus (CMV) represents a significant challenge in patients with hematological malignancies (HM), specifically those who undergo hematopoietic cell transplant (HCT). Clinical outcomes are worse in patients with end-organ disease or refractory/resistant (R/R) CMV infections (CMVi). Maribavir was FDA-approved in November 2021 for the treatment of R/R CMVi, however data on real-world experience with maribavir is limited. Our aim was to describe our institutions’ experience and 30-day outcomes of patients with CMVi treated with maribavir following the first year after approval.

**Figure 1a:**
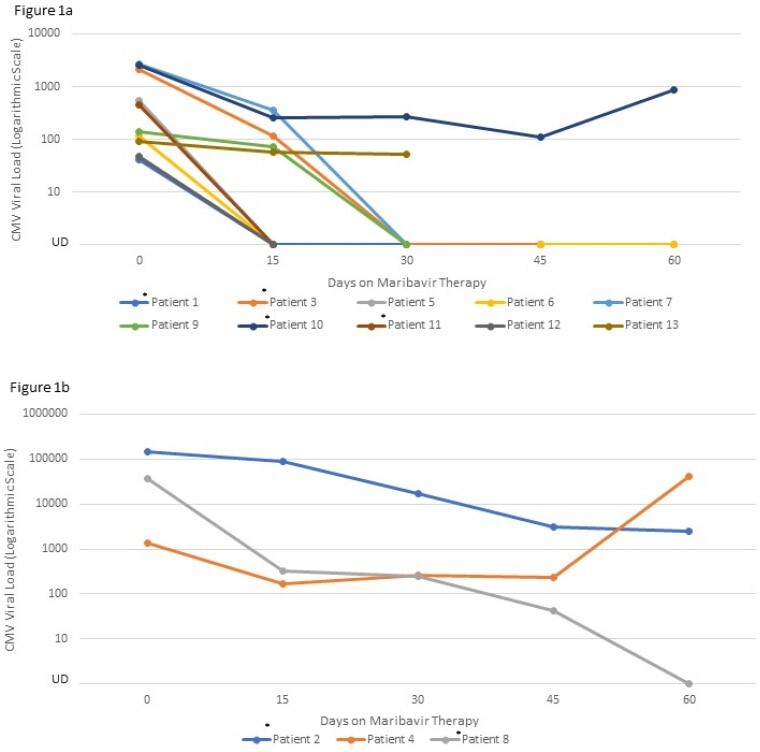
CMV viral load while on Maribavir for patients with viral load<10,000 IU/mL; Figure 1b: CMV viral load while on Maribavir for patients with viral load>10,000 IU/mL *Patients who received combination therapy with maribavir (Patients 1, 2, 3, 8, 10, 11) Abbreviation: UD (undetectable)

**Methods:**

We retrospectively reviewed all patients who received maribavir at our center between November 2021 and December 2022. Data on patient’s demographics, transplant history, and CMV management data were collected. Our primary outcome of interest was resolution of CMV viremia while on therapy and 30-day mortality. Resolution of infection was defined as having achieved sustained undetectable CMV viral load while on maribavir.

**Figure 2a:**
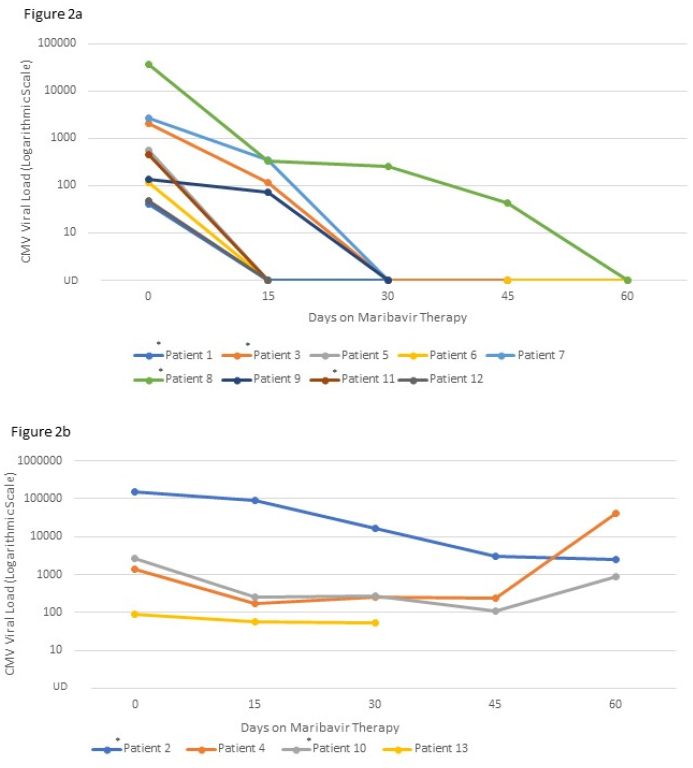
CMV viral load while on Maribavir in patients with resolution of CMV infection; Figure 2b: CMV viral load while on Maribavir in patients with non-resolution of CMV infection *Patients who received combination therapy with maribavir (Patients 1, 2, 3, 8, 10, 11) Abbreviation: UD (undetectable)

**Results:**

A total of 13 patients received maribavir at our institution during the study period; 11 were HCT recipients and 2 were patients with HM but no history of HCT (Table 1). Most patients received maribavir due to refractory CMV (53.8%); of those 2 patients had resistant CMV prior to maribavir (H520Q in UL97; C325Y in UL56), and 46.2% were switched to maribavir due to CMV therapy-related toxicities. Median duration of maribavir was 58 days. Six patients (46.2%) received combination therapy: 5 with foscarnet and 1 with leflunomide. Only 1 case progressed to CMV end-organ disease while on maribavir (Table 2). Notably, 1 patient developed maribavir resistance (C480F) and another developed UL97 polymorphism while on the antiviral (Table 2). Nine patients (69%) had resolution of CMVi while on maribavir (Table 2, Figure 2a). Seven patients had died at last follow-up with a 30-day mortality of 7.7% .

**Table 1:**
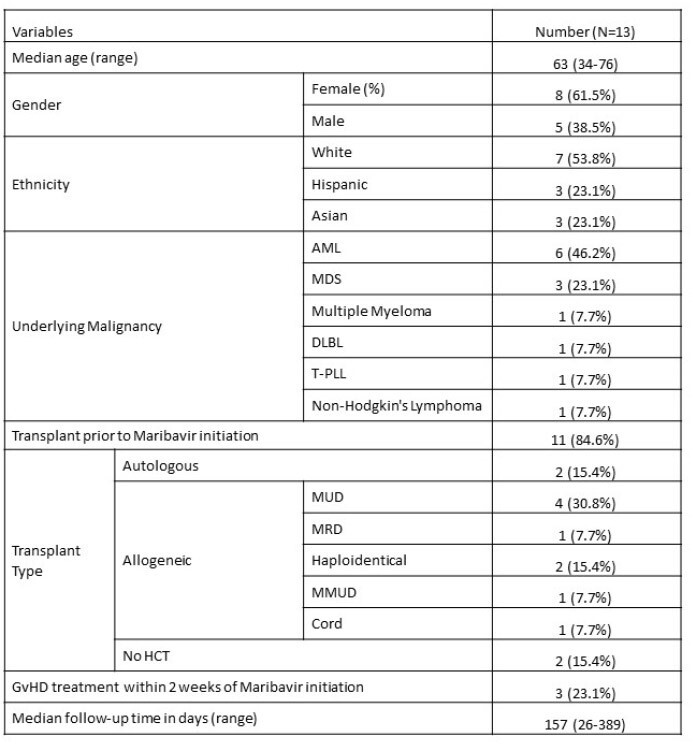
Patient and Transplant Characteristics Abbreviations: AML (acute myeloid leukemia); MDS (myelodysplastic syndrome); DLBL (diffuse large B-cell lymphoma); T-PLL (T-cell prolymphocytic leukemia); MUD (matched unrelated donor); MRD (matched related donor); MMRD (mismatched related donor); MMUD (mismatched unrelated donor); GVHD (graft-versus-host disease); HCT (hematopoietic cell transplant)

**Conclusion:**

Maribavir may provide an attractive alternative to treat R/R CMV infections or to avoid serious toxicities from currently available anti-CMV agents. On-treatment resistance is a concern and should be monitored. Larger studies are needed to evaluate long-term outcomes of patients treated with maribavir.

**Table 2:**
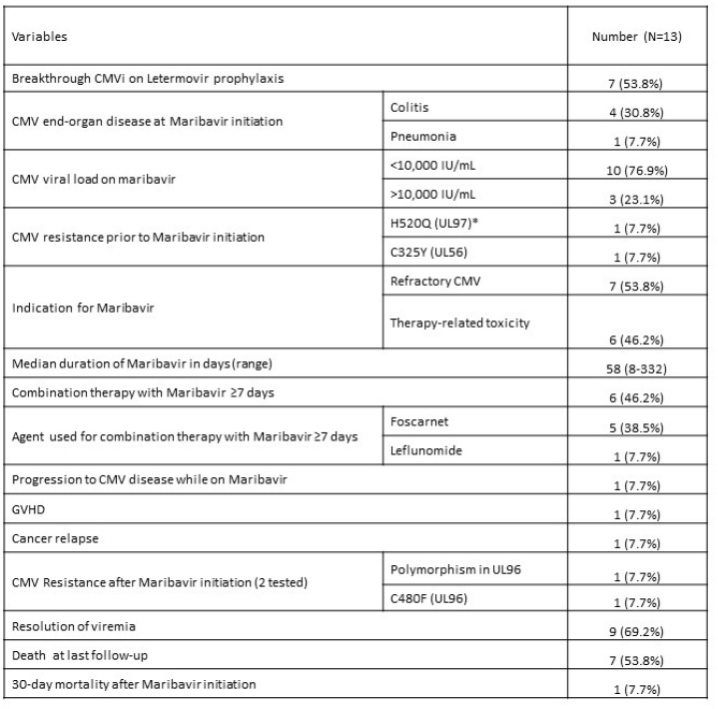
CMV- and Maribavir-Related Outcomes Abbreviations: CMVi (CMV infection); GVHD (graft-versus-host disease); IU (international units); mL (milliliters)

*H520Q confers resistance to ganciclovir but not to maribavir

**Disclosures:**

**Fareed Khawaja, MBBS**, Eurofins Viracor: Grant/Research Support|Symbio: Grant/Research Support **Roy F. Chemaly, MD/MPH**, AiCuris: Advisor/Consultant|AiCuris: Grant/Research Support|Ansun Pharmaceuticals: Advisor/Consultant|Ansun Pharmaceuticals: Grant/Research Support|Astellas: Advisor/Consultant|Eurofins-Viracor: Grant/Research Support|InflaRX: Advisor/Consultant|Janssen: Advisor/Consultant|Karius: Advisor/Consultant|Karius: Grant/Research Support|Merck/MSD: Advisor/Consultant|Merck/MSD: Grant/Research Support|Moderna: Advisor/Consultant|Oxford Immunotec: Advisor/Consultant|Oxford Immunotec: Grant/Research Support|Roche/Genentech: Advisor/Consultant|Roche/Genentech: Grant/Research Support|Shinogi: Advisor/Consultant|Takeda: Advisor/Consultant|Takeda: Grant/Research Support|Tether: Advisor/Consultant

